# NAP and D-SAL: neuroprotection against the β amyloid peptide (1–42)

**DOI:** 10.1186/1471-2202-9-S3-S3

**Published:** 2008-12-10

**Authors:** Illana Gozes, Inna Divinski, Inbar Piltzer

**Affiliations:** 1 Department of Human Molecular Genetic and Biochemistry, Sackler School of Medicine, Tel Aviv University, Einstein Street, Tel Aviv 69978, Israel

## Abstract

**Introduction:**

NAP (Asn-Ala-Pro-Val-Ser-Ile-Pro-Gln, single amino acid letter code, NAPVSIPQ), an eight amino acid neuroprotective peptide derived from activity-dependent neuroprotective protein (ADNP), exhibits some structural similarity to activity-dependent neurotropic factor-9 (ADNF-9; Ser-Alal-Leu-Leu-Arg-Ser-Ile-Pro-Ala, SALLRSIPA). Both peptides are also active in the all D-amino acid conformation, termed D-NAP and D-SAL. Original results utilizing affinity chromatography coupled to mass spectrometry identified tubulin, the subunit protein of microtubules, as the major NAP-associating protein in brain. The NAP-tubulin association was found to be diminished in the presence of ADNF-9, D-NAP, and D-SAL, suggesting a common target of neuroprotection. The β amyloid peptide interacts with microtubules, and previous studies have demonstrated protection against β amyloid (25–35) toxicity by NAP and ADNF-9. NAP also inhibits β amyloid (25–35 and 1–40) aggregation.

**Methods:**

Cerebral cortical cultures derived from newborn rats were used in neuronal survival assays to test the activity of both NAP and D-SAL against the major Alzheimer's disease toxic peptide β amyloid (1–42).

**Results:**

NAP and D-SAL protected cerebral cortical neurons against the major Alzheimer's disease toxic peptide β amyloid (1–42). Maximal protection of both peptides was observed at concentrations of 10^-15 ^to 10^-10 ^mol/l.

**Conclusion:**

These findings, together with those of previous *in vivo *studies conducted in relevant Alzheimer's disease models, pave the path to drug development. Bioavailability studies indicated that NAP penetrates cells and crosses the blood-brain barrier after nasal or systemic administration. Phase II clinical trials of NAP are currently in progress by Allon Therapeutics Inc.

## Introduction

### ADNP and ADNF

NAP (Asn-Ala-Pro-Val-Ser-Ile-Pro-Gln, single amino acid letter code, NAPVSIPQ, an eight amino acid neuroprotective peptide) is derived from activity-dependent neuroprotective protein (ADNP) [1,2], a protein that differentially interacts with chromatin to regulate genes that are essential for brain formation and embryogenesis [3-5]. Furthermore, recombinant ADNP is neuroprotective *in vitro *against severe oxidative stress and neurotoxicity associated with the Alzheimer's disease neurotoxin β amyloid peptide (25–35) [6]. ADNP synthesis and secretion is induced by the neuroprotective vasoactive intestinal peptide [1,7]. Activity-dependent neuroprotective factor (ADNF) was isolated from conditioned medium of astrocytes treated with vasoactive intestinal peptide that, in turn, was initially found to be associated with embryonic development and brain protection [8-11]. The active core of ADNF, namely ADNF-9 (SALLRSIPA), exhibits structural and functional similarities with NAP [1,12,13].

### NAP and ADNF-9: protection against β amyloid toxicity

A comprehensive review detailing NAP neuroprotective activity and clinical development [14] and a short review detailing NAP neurotropism *in vitro *were recently published [15]. Furthermore, NAP was found to enhance neurodevelopment of newborn apolipoprotein E-deficient mice subjected to hypoxia, suggesting neurotropic activity *in vivo *[16]. NAP was initially discovered to protect against β amyloid (amino acids 25–35) toxicity in rat cerebral cortical neurons seeded on a bed of astrocytes, and these studies were extended to show that NAP protected against β amyloid (25–35) in neuronal enriched cultures [1,17]. The primary structure of NAP includes two prolines that confer β sheet breaking characteristics, and NAP was shown to decrease the aggregation of the β amyloid peptide (25–35 and 1–40) [18].

The function and properties of ADNF-9 were recently reviewed [11]. Regarding Alzheimer's disease, ADNF-9 protects against the toxicity of β amyloid peptide (25–35) [9] and (1–42) [19]. In addition, primary hippocampal neurons from presenilin 1 mutant knock-in mice, exhibiting increased production of amyloid β peptide 42–43 and increased vulnerability to excitotoxicity, were protected by pretreatment with ADNF-9 [20].

The all D-amino acid analogs of NAP and ADNF-9 (D-NAP and D-SAL, respectively) have both been found to exhibit neuroprotective activity [21]. Here, we present data on the novel finding that NAP and D-SAL also protect against β amyloid (1–42) toxicity.

## Materials and methods

### Materials

The octapeptides NAP and D-SAL were synthesized by Professor M Fridkin and Ms S Rubinraut at the Department of Organic Chemistry of the Weizmann Institute of Science (Rehovot, Israel) and Bachem (Torrance, CA, USA).

All peptides were dissolved in distilled sterile water to a concentration of 1 mmol/l and then diluted in water in 1:10 steps down to the required concentration. The β amyloid peptide (1–42) was obtained from American Peptides Company (Sunnyvale, CA, USA).

### Cell cultures and neuronal survival

Cerebral cortical cultures derived from newborn rats were used for neuron survival assays. For mixed neuroglial cultures, neurons (300,000 cells/35 mm dish) were seeded on 8-day-old astrocytes prepared as described [1,21]. Cells were allowed to grow for 1 week at 37°C (10% carbon dioxide) before experiments were performed. Four days after neuronal plating, cultures were given their respective treatment and assayed for neuronal survival after an additional 5-day incubation period.

### Neuronal cell counts

The culture medium was removed and cells were washed twice with phosphate buffered saline. A volume of 1.5 ml of 3% gluteraldehyde (Fluka Biochemika, Steinheim, Germany) in 0.1 mol/l cacodylic acid (pH 7.2; Fluka Biochemika) was added for 2 hours. The cells were then washed with phosphate buffered saline and 2 ml of 0.15 mol/l cacodylic acid (pH 7.2) was added. Neuronal identity was established by morphological criteria using an Olympus CK2 light microscope (Olympus, Tokyo, Japan) with a 40× lens. Fifty fields were counted per dish [17].

## Results

**Figure 1:**
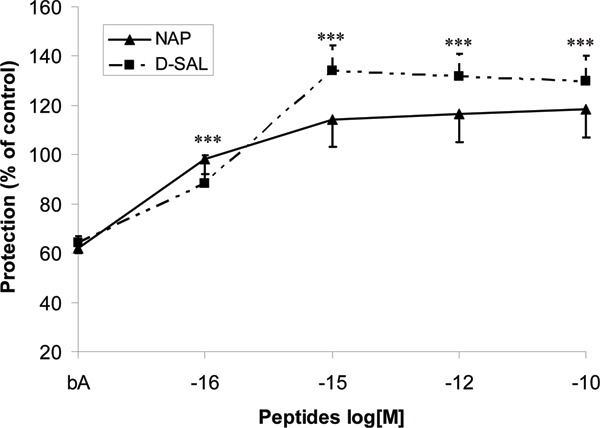
The eight and nine amino acid peptides (NAP and D-SAL, respectively) provide neuroprotection against β amyloid (1–42). Mixed neuroglial cultures were exposed to 2.5 μmol/l β amyloid peptide (1–42) for 5 days, resulting in about 40% cell death. The respective peptides were added together with the toxin at indicated concentrations (10^-16 ^mol/l, 10^-15 ^mol/l, 10^-12 ^mol/l, and 10^-10 ^mol/l). Experiments were repeated at least three times. Results were normalized by untreated cells. ***P < 0.001, cells treated with either NAP or D-SAL versus β amyloid alone (without peptide treatment). D-SAL, D-amino acid analog of activity-dependent neuroprotective factor 9; NAP, Asn-Ala-Pro-Val-Ser-Ile-Pro-Gln, single amino acid letter code, NAPVSIPQ, an eight amino acid neuroprotective peptide derived from activity-dependent neuroprotective protein.

The number of surviving neurons was assessed in cerebral cortical cultures derived from newborn rats using 2.5 μmol/l of β amyloid peptide (1–42), a toxin associated with Alzheimer's disease. NAP and D-SAL were used at the following concentrations: 10^-16 ^mol/l, 10^-15 ^mol/l, 10^-12 ^mol/l, and 10^-10 ^mol/l. The peptides protected against neurotoxicity associated with the β amyloid peptide (1–42; *P *< 0.001). Maximal protection of both peptides was observed at concentrations of 10^-15 ^mol/l to 10^-10 ^mol/l (Figure 1). No differences were observed between NAP and D-SAL in terms of neuroprotection. Cell counts totaled more than 100% of control. This may be because the treatment prevented neuronal cell death that occurred naturally in the cultures (10% to 20%), as previously observed [1].

## Discussion

### Interactions of NAP and D-SAL with microtubules

The β amyloid peptide (1–42) has been shown to aggregate into oligomers along microtubules of neuronal processes, both in Tg2576 mice and human Alzheimer's disease brain [22]. In triple transgenic Alzheimer's disease mice expressing three mutant transgenes (amyloid precursor protein [double Swedish, K670M/N671L], presenilin 1 [M146V], and tau [P301L]) [23], β amyloid accumulation occurs before tau hyperphosphorylation. These findings have been suggested to imply, at least in part, a possible cause and effect relationship between β amyloid and tau pathology. A main target of NAP in neuroprotection appears to be tubulin, which is the major subunit of microtubules. Affinity chromatography studies utilizing immobilized NAP identified tubulin as the NAP associating protein [24]. D-SAL diminished tubulin association with the NAP affinity column, suggesting an interaction of tubulin with D-SAL as well [25]. Tubulin antibodies identified NAP interaction with the neuron-specific tubulin subunit β III, as well as with astrocyte tubulin [26]. Further studies showed that although vincristine (which enhances microtubule depolymerization and crystal formation) did not diminish the interaction of NAP with tubulin, paclitaxel (a microtubule stabilizing agent) did diminish this interaction [26]. NAP accelerated microtubule formation after nocodazole depolymerization in neurons [27]. Furthermore, NAP prevented microtubule disassembly caused by zinc chloride intoxication in astrocytes [24] and in neurons [26].

The temporal changes in microtubule assembly that followed NAP treatment in cells paralleled changes in phosphorylation of the tubulin-associated protein, tau [28]. This finding suggests that NAP regulates tau phosphorylation alongside microtubule dynamics and protects astrocytes and neurons. In the triple transgenic Alzheimer's disease mouse model, NAP reduced tau hyperphosphorylation [29]. Because tau hyperphosphorylation has been associated with major neurodegenerative diseases, including Alzheimer's disease, NAP holds promise as a neuroprotective/neurotropic drug candidate.

### Bioavailability

#### Cellular bioavailability

Fluoresceine-labeled NAP was detected in the intracellular milieu of neurons and astrocytes. In astrocytes, labeled NAP was found even when incubated at 4°C and in conditions of low pH, indicating membrane permeability. Furthermore, comparison of NAP with known membrane permeating peptides has revealed that NAP possesses a membrane permeation structure [24]. Because both NAP and ADNF-9 are active in their all D-amino acid conformation, these peptides were suggested to be mechanistically achiral [21]. It is our working hypothesis that NAP interacts with intracellular tubulin to enhance microtubule polymerization and provide cellular protection. However, NAP interacts with specific tubulin subunits and does not provide protection to all cells. Indeed, NAP did not protect fibroblast-like cells, but did protect neuronal-like cells against oxidative stress [26]. These results are in accordance with our original studies suggesting that NAP selectively interacts with brain specific tubulin subunits that are associated with multiple tubulin functions [30-32].

#### Brain bioavailability and clinical development

The pharmacodynamic compartment for NAP is the brain or the central nervous system. When NAP is administered it must be able to reach this target compartment at pharmacologically active concentrations. As shown in Figure 1, NAP exhibits an *in vitro *potency of about 10^-15 ^mol/l. Preclinical and phase I clinical experiments demonstrated that intranasal administration of NAP to rat, dog, or human results in measurable plasma levels [14]. After intranasal administration of [^3^H]NAP to rats, radioactivity was detected in the blood and was also measured in the various organs of the body [33]. Intact peptide was identified in the rat cortex 30 and 60 minutes after intranasal administration. In the permanent middle cerebral artery occlusion rat model, intravenous administration of radioactive NAP resulted in measurable levels in the cerebellum and cortex 15 minutes after injection and was maintained for at least 30 minutes in ischemic tissue [34]. Liquid chromatography mass spectrometry assays in rats and dogs corroborated and extended these results. Recent data from a pharmacokinetic study conducted in rats suggested a correlation between plasma and cerebrospinal fluid levels of NAP administered by intravenous injection. After intranasal administration in the rat, appeared NAP rapidly in plasma and the kinetics of appearance in cerebrospinal fluid (T_max_) appeared to lag plasma T_max _[14,35]. It is therefore likely that access to the brain is via the circulation for both intravenous and intranasal routes.

## Conclusion

NAP and D-SAL protect against the Alzheimer's disease neurotoxin β amyloid peptide (1–42), which suggests potential treatments for Alzheimer's disease pathology. This finding, together with previous findings including animal efficacy and bioavailability studies, steer these compounds toward clinical development. Allon Therapeutics Inc. [36] recently released top-line results of a phase IIa clinical trial showing that its drug AL-108 (the intranasal formulation of NAP) has a positive impact on memory function in patients with amnestic mild cognitive impairment, which is a precursor to Alzheimer's disease.

## List of abbreviations used

ADNF: activity-dependent neuroprotective factor; ADNP: activity-dependent neuroprotective protein; NAP: Asn-Ala-Pro-Val-Ser-Ile-Pro-Gln (single amino acid letter code, NAPVSIPQ).

## Competing interests

NAP and D-SAL are under patent protection and licensed for clinical development to Allon Therapeutics Inc. IG serves as the Chief Scientific Officer of Allon Therapeutics Inc.

## Authors' contributions

IG conceived of the study, and participated in its design and coordination. IN and IP carried out the experiments. IG drafted the manuscript. All authors participated in writing, and read and approved the final manuscript.

## References

[B1] Bassan M, Zamostiano R, Davidson A, Pinhasov A, Giladi E, Perl O, Bassan H, Blat C, Gibney G, Glazner G, Brenneman DE, Gozes I (1999). Complete sequence of a novel protein containing a femtomolar-activity-dependent neuroprotective peptide. J Neurochem.

[B2] Gozes I (2007). Activity-dependent neuroprotective protein: from gene to drug candidate. Pharmacol Ther.

[B3] Pinhasov A, Mandel S, Torchinsky A, Giladi E, Pittel Z, Goldsweig AM, Servoss SJ, Brenneman DE, Gozes I (2003). Activity-dependent neuroprotective protein: a novel gene essential for brain formation. Brain Res Dev Brain Res.

[B4] Mandel S, Rechavi G, Gozes I (2007). Activity-dependent neuroprotective protein (ADNP) differentially interacts with chromatin to regulate genes essential for embryogenesis. Dev Biol.

[B5] Mandel S, Gozes I (2007). Activity-dependent neuroprotective protein constitutes a novel element in the SWI/SNF chromatin remodeling complex. J Biol Chem.

[B6] Steingart RA, Gozes I (2006). Recombinant activity-dependent neuroprotective protein protects cells against oxidative stress. Mol Cell Endocrinol.

[B7] Furman S, Steingart RA, Mandel S, Hauser JM, Brenneman DE, Gozes I (2004). Subcellular localization and secretion of activity-dependent neuroprotective protein in astrocytes. Neuron Glia Biol.

[B8] Gozes I, Davidson A, Gozes Y, Mascolo R, Barth R, Warren D, Hauser J, Brenneman DE (1997). Antiserum to activity-dependent neurotrophic factor produces neuronal cell death in CNS cultures: immunological and biological specificity. Brain Res Dev Brain Res.

[B9] Brenneman DE, Gozes I (1996). A femtomolar-acting neuroprotective peptide. J Clin Invest.

[B10] Gozes I, Brenneman DE (1996). Activity-dependent neurotrophic factor (ADNF). An extracellular neuroprotective chaperonin?. J Mol Neurosci.

[B11] Gozes I, Vulih I, Spivak-Pohis I, Furman S, Henderson B, Pockley AG (2005). Neuroendocrine aspects of the molecular chapernoes ADNF and ADNP. Molecular Chaperones and Cell Signalling.

[B12] Gozes I, Bassan M, Zamostiano R, Pinhasov A, Davidson A, Giladi E, Perl O, Glazner GW, Brenneman DE (1999). A novel signaling molecule for neuropeptide action: activity-dependent neuroprotective protein. Ann N Y Acad Sci.

[B13] Brenneman DE, Hauser J, Neale E, Rubinraut S, Fridkin M, Davidson A, Gozes I (1998). Activity-dependent neurotrophic factor: structure-activity relationships of femtomolar-acting peptides. J Pharmacol Exp Ther.

[B14] Gozes I, Morimoto BH, Tiong J, Fox A, Sutherland K, Dangoor D, Holser-Cochav M, Vered K, Newton P, Aisen PS, Matsuoka Y, van Dyck CH, Thal L (2005). NAP: research and development of a peptide derived from activity-dependent neuroprotective protein (ADNP). CNS Drug Rev.

[B15] Gozes I, Spivak-Pohis I (2006). Neurotrophic effects of the peptide NAP: a novel neuroprotective drug candidate. Curr Alzheimer Res.

[B16] Rotstein M, Bassan H, Kariv N, Speiser Z, Harel S, Gozes I (2006). NAP enhances neurodevelopment of newborn apolipoprotein E-deficient mice subjected to hypoxia. J Pharmacol Exp Ther.

[B17] Zemlyak I, Furman S, Brenneman DE, Gozes I (2000). A novel peptide prevents death in enriched neuronal cultures. Regul Pept.

[B18] Ashur-Fabian O, Segal-Ruder Y, Skutelsky E, Brenneman DE, Steingart RA, Giladi E, Gozes I (2003). The neuroprotective peptide NAP inhibits the aggregation of the beta-amyloid peptide. Peptides.

[B19] Hashimoto Y, Kaneko Y, Tsukamoto E, Frankowski H, Kouyama K, Kita Y, Niikura T, Aiso S, Bredesen DE, Matsuoka M, Nishimoto I (2004). Molecular characterization of neurohybrid cell death induced by Alzheimer's amyloid-beta peptides via p75NTR/PLAIDD. J Neurochem.

[B20] Guo Q, Sebastian L, Sopher BL, Miller MW, Glazner GW, Ware CB, Martin GM, Mattson MP (1999). Neurotrophic factors [activity-dependent neurotrophic factor (ADNF) and basic fibroblast growth factor (bFGF)] interrupt excitotoxic neurodegenerative cascades promoted by a PS1 mutation. Proc Natl Acad Sci USA.

[B21] Brenneman DE, Spong CY, Hauser JM, Abebe D, Pinhasov A, Golian T, Gozes I (2004). Protective peptides that are orally active and mechanistically nonchiral. J Pharmacol Exp Ther.

[B22] Takahashi RH, Almeida CG, Kearney PF, Yu F, Lin MT, Milner TA, Gouras GK (2004). Oligomerization of Alzheimer's beta-amyloid within processes and synapses of cultured neurons and brain. J Neurosci.

[B23] Oddo S, Caccamo A, Shepherd JD, Murphy MP, Golde TE, Kayed R, Metherate R, Mattson MP, Akbari Y, LaFerla FM (2003). Triple-transgenic model of Alzheimer's disease with plaques and tangles: intracellular Abeta and synaptic dysfunction. Neuron.

[B24] Divinski I, Mittelman L, Gozes I (2004). A femtomolar acting octapeptide interacts with tubulin and protects astrocytes against zinc intoxication. J Biol Chem.

[B25] Holtser-Cochav M, Divinski I, Gozes I (2006). Tubulin is the target binding site for NAP-related peptides: ADNF-9, D-NAP, and D-SAL. J Mol Neurosci.

[B26] Divinski I, Holtser-Cochav M, Vulih-Schultzman I, Steingart RA, Gozes I (2006). Peptide neuroprotection through specific interaction with brain tubulin. J Neurochem.

[B27] Gozes I, Divinski I (2007). NAP, a neuroprotective drug candidate in clinical trials, stimulates microtubule assembly in the living cell. Curr Alzheimer Res.

[B28] Gozes I, Divinski I (2004). The femtomolar-acting NAP interacts with microtubules: Novel aspects of astrocyte protection. J Alzheimers Dis.

[B29] Matsuoka Y, Jouroukhin Y, Gray AJ, Ma L, Hirata-Fukae C, Li HF, Feng L, Lecanu L, Walker BR, Planel E, Arancio O, Gozes I, Aisen PS (2008). A neuronal microtubule-interacting agent, NAPVSIPQ, reduces tau pathology and enhances cognitive function in a mouse model of Alzheimer's disease. J Pharmacol Exp Ther.

[B30] Gozes I, Littauer UZ (1978). Tubulin microheterogeneity increases with rat brain maturation. Nature.

[B31] Gozes I, Sweadner KJ (1981). Multiple tubulin forms are expressed by a single neurone. Nature.

[B32] Gozes I, Barnstable CJ (1982). Monoclonal antibodies that recognize discrete forms of tubulin. Proc Natl Acad Sci USA.

[B33] Gozes I, Giladi E, Pinhasov A, Bardea A, Brenneman DE (2000). Activity-dependent neurotrophic factor: intranasal administration of femtomolar-acting peptides improve performance in a water maze. J Pharmacol Exp Ther.

[B34] Leker RR, Teichner A, Grigoriadis N, Ovadia H, Brenneman DE, Fridkin M, Giladi E, Romano J, Gozes I (2002). NAP, a femtomolar-acting peptide, protects the brain against ischemic injury by reducing apoptotic death. Stroke.

[B35] Morimoto BH, de Lannoy I, Liu X, Gien B, Yang Y, Fox A, Tiong J, Gozes I (2006). Pharmacokinetics of the neuroprotective peptide, NAPVSIPQ in serial samples of plasma and CSF after intravenous or intranasal administration. Drug Metab Rev.

[B36] Allon Therapeutics Inc. http://www.allontherapeutics.com.

